# Retrospective Cohort Study of Transgender Adolescents at Strasbourg University Hospital

**DOI:** 10.3390/children13060789

**Published:** 2026-06-06

**Authors:** Camille Schunder, Agnès Gras-Vincendon, François Brezin

**Affiliations:** 1 Pediatric Endocrinology and Diabetology Unit, Department of Pediatrics 1, University Hospitals of Strasbourg, 1 Avenue Molière, 67000 Strasbourg, France; camille.schunder@chru-strasbourg.fr; 2 Department of Child and Adolescent Psychiatry, University Hospitals of Strasbourg, 1 Place de l’Hôpital, 67000 Strasbourg, France; agnes.gras@chru-strasbourg.fr

**Keywords:** transidentity, transgender, FtM, MtF, AFAB, AMAB, gender incongruence, gender dysphoria, GAHT, puberty blockers

## Abstract

**Highlights:**

• **Main findings**: Only 115 patients were followed in our referral center, which serves a population of nearly 2 million inhabitants (noting that among the 115, 16 adolescents lived outside the region). As reported in other cohorts, we found a very high rate of psychiatric co-occurrences in these youths. Among the 115 patients, only 36.5% received hormone therapy before the age of 18, after a median follow-up duration of 15 months. Surprisingly, in our cohort, transition pathways appear to differ according to the adolescent’s type of schooling. No official definition of retransition has been established by professional societies. In our cohort, the rate of retransition ranged from 0% to 6%, depending on the definition applied. Nevertheless, no adolescent retransitioned to their sex assigned at birth, nor did any express regret after initiating gender-affirming hormone therapy.
• **Implications of the main findings**: The number of transgender youths followed in our center remains relatively low compared with the population of our region. This observation is far from the so called “epidemic of trans children” sometimes portrayed in certain media. Nevertheless, these young people present numerous psychiatric co-occurrences and require appropriate, structured, and compassionate support. It is essential that data from the various specialized centers continue to be published. Improved clinical and epidemiological knowledge will help enhance the care provided to these youths, based on scientifically grounded recommendations. The observation that the transition pathways and modalities of gender transition differ according to the type of schooling has, to our knowledge, never been described and deserves further investigation in future studies. Moreover, it seems important for professional societies to address the question of how “retransition” should be defined, in order to establish a shared definition that would allow studies to be compared more reliably.

**Abstract:**

**Introduction:** Medical care for transgender minors is understudied, largely because these forms of care are relatively recent. The primary objective of this work was to describe the cohort of transgender adolescents who initiated follow-up at the Strasbourg University Hospital before the age of 18, whether or not they began hormone therapy prior to reaching adulthood. **Method**: This was an observational, retrospective, single-center, descriptive study conducted among adolescents who had attended at least one consultation in our center before the age of 18 between January 2017 and March 2024. **Results**: Our population consisted of 115 patients predominantly made up of transmasculine (AFAB) adolescents (68%). Compared with the general population, we observed significantly higher rates of psychiatric co-occurrences, autism spectrum disorder (ASD), and attention-deficit/hyperactivity disorder (ADHD). Only 46.1% initiated gender-affirming hormone therapy (GAHT) in our cohort, and just 34.8% before age 18. A total of 6% of adolescents received puberty blockers as monotherapy. The mean age at GAHT initiation was 16.99 years. Transition pathways appear to differ according to the adolescent’s type of schooling. The rate of retransition/treatment interruption in our sample ranged from 0% to 6.1%, depending on the criteria applied. We did not identify any adolescent who retransitioned to their sex assigned at birth after starting GAHT by the end of the data collection. **Discussion**: The high prevalence of psychiatric co-occurrences raises important questions regarding how to improve care for these adolescents. The predominance of AFAB adolescents similarly prompts reflection on the barriers that transfeminine adolescents may face when seeking to transition before adulthood. In addition, the substantial number of adolescents presenting with ASD or ADHD underscores the need for particular vigilance regarding their specific needs and overall well-being. Finally, the variability in retransition rates depending on the criteria applied highlights the absence of a consensual definition, which limits the comparability and validity of existing studies. **Conclusions**: Long-term prospective studies are needed to objectively demonstrate the effectiveness of current transition pathways. Academic research in this field should be strengthened, along with the development of larger prospective datasets, to improve the overall health of this population.

## 1. Introduction

A transgender person is an individual who experiences gender incongruence. According to the ICD-11 definition, gender incongruence is a marked and persistent mismatch between an individual’s experienced gender and their assigned sex. Unlike the older DSL-IV designation “gender dysphoria”, it is classified as a condition related to sexual health rather than a mental disorder, reflecting current international consensus. The concept focuses on the incongruence itself, not on associated distress, and is used to guide access to gender-affirming care in many clinical settings. Prevalence estimates vary widely depending on the methodology and definitions used across studies. According to the most recent literature reviews, prevalence appears to range from 0.3% to 0.5% in adults and from 1.2% to 2.7% in adolescents [1].

The United States [2], Canada [3], Australia [4], the Netherlands [5], and the United Kingdom [6] were the pioneering countries in the development of medical care for pediatric transgender populations in Western countries. These care models subsequently expanded across most European countries, although with considerable variability in practices and regulatory frameworks [7]. In France, specialized services began to emerge from 2013 onward [8].

All countries in which dedicated clinical services for transgender adolescents were established consistently reported a progressive increase in referral rates [2,8,9]. This raises the need to distinguish between a genuine increase in pediatric transgender cases (and, by extension, to consider the potential causes of such an increase) and a possible recording bias resulting from improved access to appropriate medical care [8].

In Strasbourg, medical support for transgender adolescents developed through a coordinated community–hospital network beginning in 2016–2017, structured around multidisciplinary team meetings (MDTs) (adult and child/adolescents psychiatrists, psychologist, adult and pediatric endocrinologist, surgeon, gynecologist, reproductive biologist, general practitioner, sociologist), held under the auspices of the Strasbourg University Hospital. The care model is based on a multiprofessional approach involving a mental health practitioner within the network, occasionally joined, when clinically indicated, by a network endocrinologist and a fertility specialist, all of whom are trained and experienced in supporting transgender individuals.

For several years, transgender adolescents were first assessed by the psychiatrists within the network, and only subsequently referred to the endocrinologist. In the absence of a dedicated pediatric endocrinology service, gender-affirming hormone therapy (GAHT) was prescribed by adult endocrinologists in the network. Access to GAHT was long restricted to adolescents aged 16 years or older and required at least one year of prior child and adolescent psychiatric follow-up. Younger adolescents were not eligible for puberty blockers.

The opening of a dedicated pediatric endocrinology consultation in 2020 enabled the Strasbourg network to join a national pediatric endocrinology network coordinated by the French Society of Pediatric Endocrinology and Diabetology [10]. This national network aims to harmonize practices across France and align them with international standards. This opening:

- Allowed younger transgender adolescents to receive puberty blockers when clinically indicated.
- Change the care pathway. Today, the first professional of the network encountered is no longer necessarily the psychiatrist. In some cases, families first contact the secretary of the pediatric endocrinologist, often following the advice of their general practitioner. The adolescent is then referred to a mental health professional at a later stage. If the family contacts both secretariats (endocrinology and psychiatry), the first professional the adolescent meets is simply the one with the earliest availability. Regarding mental health care, the adolescent will first meet either the psychiatrist or the psychologist, depending on the needs identified during the initial history-taking by the endocrinologist (if seen first) or by the referring general practitioner.
- The minimum age for initiating GAHT has been lowered to 15 years old, although international guidelines now emphasize maturity-based criteria rather than strict age thresholds.
- Henceforth, the duration and frequency of follow-up are individualized and take into account the needs expressed by the adolescent and their parents, including the degree of family acceptance, any prior psychological or medical care, age, maturity, and the level of psychological distress.

The objectives of this follow-up are multifaceted [1,7,10]:

- To support the adolescent in exploring their gender identity without exerting influence in any direction.
- To confirm that the adolescent’s situation meets the diagnostic criteria for gender incongruence, to identify any associated somatic, psychiatric, and/or neurodevelopmental co-occurrences, and, when present, to ensure their appropriate management.
- To characterize the nature of the request (monitoring of co-occurring conditions? Psychological support only? Desire for social transition only? Request for hormonal transition?), to ascertain its persistence, to assess the degree of family consensus regarding the request, and to propose solutions that best accommodate all parties while maintaining a care approach centered on the adolescent.
- To assess the adolescent’s capacity for discernment and their ability to provide free and informed consent to the proposed care, particularly regarding medical and hormonal treatments.
- To provide the adolescent and their legal guardians with the most complete information possible on the various modalities of transition, hormonal treatments, the irreversibility of certain effects, potential adverse outcomes, and the possibility of discontinuing the process at any time, whether before or after initiating any treatment. A consultation with a specialist physician is systematically offered to discuss options for gamete preservation.
- To perform a preliminary medical evaluation to rule out any contraindications (particularly somatic) to gender-affirming hormone therapy (GAHT) and to identify any specific monitoring needs.
- When GAHT is initiated, the clinician ensures that the adolescent provides informed consent and that their legal guardians give their approval. Rather than simply signing a form, all parties handwrite their consent in full, following a template established by the team.
- A dedicated MDT reviews the files of the minors concerned, including proposed hormonal treatments, every three months. When required, the MDT seeks input from other medical specialties and/or refers cases to additional specialized MDTs.

Several pediatric cohort studies have been published internationally [11,12,13,14,15,16,17], but only one French cohort [8]. Our work therefore represents the second published pediatric cohort from France. The primary objective of our study was to describe the cohort of transgender adolescents who initiated follow-up for gender incongruence at Strasbourg University Hospital before the age of 18, whether or not they received GAHT prior to reaching adulthood.

## 2. Materials and Methods

### 2.1. Population

This study is an observational, retrospective, single-center, descriptive analysis based on the medical records of transgender patients (ICD-10 code F64.0, F64.2, F64.8 and F64.9) who attended at least one consultation before the age of 18 in the Child and Adolescent Psychiatry and/or Pediatric Endocrinology departments of Strasbourg University Hospital. Data collection covered the period from January 2017 to March 2024. Ethical approval was obtained on 15 May 2025. After reviewing all clinical files of adolescents seen in pediatrics and/or child psychiatry for questions related to gender identity at Strasbourg University Hospital, a total of 115 patients were included. Exclusion criteria were: age over 18 at the time of the first consultation or insufficiently documented records.

### 2.2. Variables Studied

We first collected data on the date of the initial consultation with the first member of the team (psychiatrist, psychologist, or endocrinologist) who was seen and who starting the trajectory, sex assigned at birth, the age at first consultation, and the type of transition underway or desired. We also recorded the ages at coming in (defined as the adolescent’s first awareness of gender incongruence), coming out (defined as the adolescent’s first disclosure of gender incongruence to another person) as reported by the adolescents, age at the change of first name on school attendance records (which is permitted in France before any official change), and age at the legal name change and schooling pathway (general education track, vocational education track, remote learning, other).

Review of child and adolescent psychiatry records allowed us to document the number of psychiatric consultations prior to the initiation of GAHT, as well as any psychiatric antecedents or co-occurring conditions diagnosed by the psychiatrist of the youth, including anxiety, depression, suicidal ideation, self-harm, suicide attempts, eating disorders, autism spectrum disorder (ASD), attention-deficit/hyperactivity disorder (ADHD), and psychosocial factor such as history of sexual violence or school bullying. Medical correspondence was used to identify the date of first contact with an endocrinologist when follow-up occurred in private practice. Data on the prevalence of psychiatric disorders in the general French population were obtained from governmental sources such as “Santé Publique France” (governmental public health statistic service), the French National Authority for Health, or published studies when available. Because prevalence estimates vary across studies, the highest reported values were retained to minimize the risk of drawing inaccurate comparisons.

Analysis of pediatric endocrinology records provided information on age at first endocrinology contact, the number of endocrinology consultations prior to GAHT initiation, the type of treatment prescribed (when applicable), the date of the MDT, and whether the adolescent had attended a fertility preservation center and subsequently undergone gamete preservation.

### 2.3. Statistical Analyses

The Chi square test was used to compare categorical variables. When observed counts in 2 × 2 tables were low (<10), Yates’ correction was applied. Student’s *t*-test was used for comparisons of means (reported with standard deviation). All medians are presented together with their first and third quartiles (Q1 and Q3). Odds ratios (ORs) with 95% confidence intervals (CI 95%) were computed to assess the magnitude and precision of associations.

## 3. Results

### 3.1. General Description of the Sample

**Figure 1:**
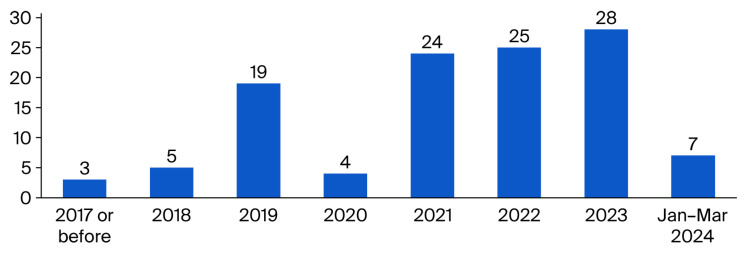
Number of new patients managed for gender identity at Strasbourg University Hospital per year.

Figure 1 shows the number of first consultations for gender incongruence among minors at Strasbourg University Hospital. This number increased between 2017 and 2021 (with a marked decrease in 2020) and has remained relatively stable since then.

**Figure 2:**
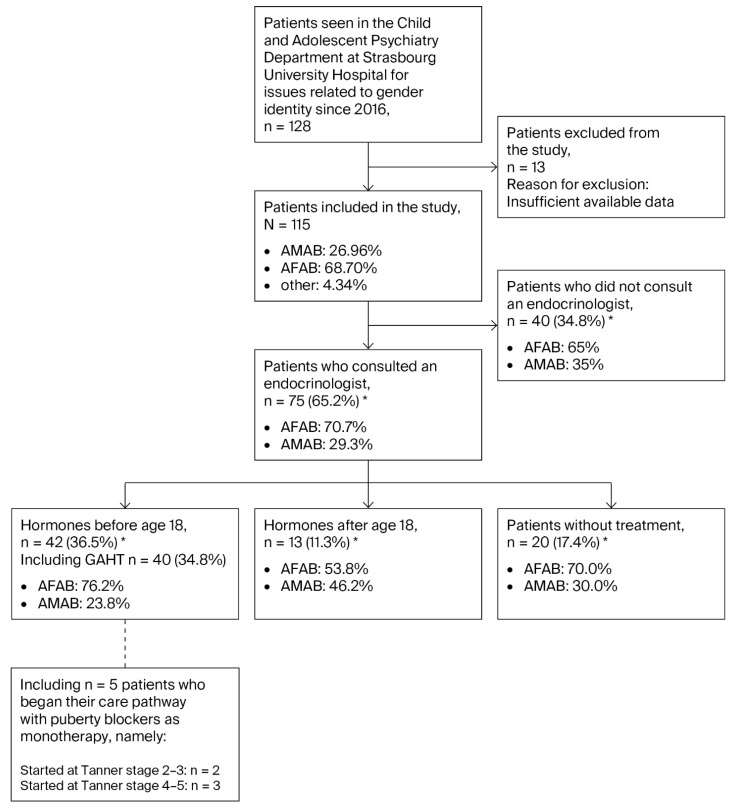
Distribution of the patients (* percentage of the included patients).

The sex ratio in our population is 2.4, with 68.7% (*n* = 79) assigned female at birth (AFAB) adolescents and 26.96% (*n* = 31) assigned male at birth (AMAB) adolescents. This sex ratio remains broadly similar across all age groups (Figure 2). In addition, 0.8% (*n* = 1) identified as non-binary, and 3% (*n* = 4) were classified as “other” because they were still questioning their gender. Overall, 85% initiated follow-up through a child and adolescent psychiatry consultation, while 15% began care in pediatric endocrinology. Ages at first consultation ranged from 6 to 17 years. The mean age was 15.11 years (±2.16), with a median of 15.55 years (Q1: 14.2; Q3: 16.5).

Because of variability and the approximate nature of these self-reported ages, coming-in and coming-out ages are presented as medians in our study. The median age of coming-in individuals was 12 years (Q1: 10; Q3: 14), and the median age of coming-out individuals was 14 years (Q1: 12; Q3: 15). The median age at coming in among AFAB individuals was 12 years (Q1: 11; Q3: 14), and the median age at coming out was 14 years (Q1: 12.5; Q3: 15). Among AMAB individuals, the median age at coming in was 11 years (Q1: 7.75; Q3: 13.13), while the median age at coming out was 14 years (Q1: 12.5; Q3: 15).

After coming out, 53 adolescents (46%) were able to change their first name on the school attendance records. A total of 38 (33%) applied for an official civil-registry name change, which was granted at a mean age of 15.9 years old. The median time between coming in and the first consultation with the team was 3 years (Q1: 2; Q3: 5), and the median time between coming out and the first consultation was 1 year (Q1: 3 months; Q3: 2 years). Regardless of sex assigned at birth, coming-in and coming-out ages were similar across the cohort.

In our study, 44% were enrolled in the general academic track, 31% in vocational education track, and 11% followed remote learning. A total of 13% were not in standard schooling: they were engaged in civic service, employed, inactive, and/or had dropped out of school.

### 3.2. Child and Adolescent Psychiatric Care

Regarding psychiatric co-occurring conditions (Table 1), 62% of transgender adolescents reported having experienced suicidal ideation, and 29% had made at least one suicide attempt. A total of 34% presented (or had previously presented) with depressive symptoms, 36% reported a history of self-harm (e.g., cutting), 52% had (or had had) an anxiety disorder, and 15% an eating disorder. A total of 7.8% of adolescents had an ASD and 13% had ADHD. Of the 115 adolescents included, a total of 96 (83.5%) had at least one psychiatric co-occurrence. When ASD and ADHD diagnoses were excluded, this number decreased to 92 individuals (80%).

Regarding other psychosocial factors, a total of 10.4% of adolescents in the cohort reported having experienced sexual violence: 11.1% among those AFAB and 8.82% among those AMAB. School bullying was another variable of interest and affected 29.6% of the sample.

We compared several mental health parameters in our cohort with those of the general population and with the findings reported by the only other French cohort Lagrange et al. These comparisons are summarized in Table 2.

### 3.3. Endocrinology Care

#### 3.3.1. Distribution of Patients

Of the 115 adolescents included, 75 (65%) consulted an endocrinologist, including 40 who were seen by a pediatric endocrinologist. The distribution of patients is summarized in Figure 2.

#### 3.3.2. Social Factors Associated with Endocrinology Care

We examined the factors associated with whether a transgender adolescent in our cohort consulted an endocrinologist and whether they received GAHT. Regarding schooling, 73% of adolescents enrolled in non-general education tracks (vocational track, remote learning, or other) consulted an endocrinologist, compared with only 55% of those in the general academic track (*p* = 0.038). Adolescents in general education track were more likely to be referred to pediatric endocrinologists (68%) than those following other types of schooling (43%) (*p* = 0.034).

Regarding the referral to an endocrinologist, there was no difference according to the type of transition (AFAB or AMAB) (*p* = 0.69).

#### 3.3.3. Initiation of Endocrinology Care

The relevant elements related to endocrine care are summarized in Table 3.

Adolescents who received endocrinology care (65%) began follow-up at a mean age of 16.04 years (±1.87) and a median age of 16.5 years (Q1: 15.1; Q3: 17.34).

Among the 75 adolescents who consulted an endocrinologist, 53 received GAHT and 5 were treated with puberty blockers.

The mean age at initiation of GAHT was 16.99 years (±0.98). A total of 40 adolescents (75.5%) began treatment before the age of 18, while 13 (24.5%) started at 18 years or older, for a total of 53 patients, representing 46% of the entire cohort. The 40 adolescents who initiated treatment while still minors accounted for 34.8% of the full sample of 115 patients.

Among these 40 adolescents:

- Twenty-six (65%) started GAHT between ages 16 and 18;
- Fourteen (35%) started GAHT between ages 15 and 16;
- No GAHT was initiated before age 15.

The median time from first contact to initiation of GAHT was approximately 15 months (Q1: 11; Q3: 21). Between this first contact and the start of GAHT, adolescents had a median of 9 child and adolescent psychiatry consultations (Q1: 7; Q3: 12) and 2 endocrinology consultations (Q1: 2; Q3: 3).

During follow-up, a consultation at the fertility preservation center was systematically offered. A total of 32 adolescents attended, and 6 of them underwent gamete preservation. Among those who received GAHT (*n* = 53), 26 (49%) had been seen at the fertility preservation center beforehand, including 20 AFAB adolescents (54% of AFAB receiving GAHT) and 6 AMAB adolescents (44% of AMAB receiving GAHT). All six adolescents who proceeded with gamete preservation were AMAB.

Initiation of GAHT was discussed in MDT for 33 of the 53 adolescents receiving GAHT, partly because 12 of them were adults at the time of initiation. Two adolescents initially received an unfavorable MDT opinion, followed by a favorable opinion upon re-evaluation. Among the 40 adolescents (68%) who initiated GAHT before 18 years old, 27 (68%) had their treatment discussed in MDT. The 13 adolescents who started GAHT before age 18 without MDT review were all followed in private adult endocrinology practices. The reasons why some cases were not reviewed in MDT are unknown, as only internal hospital records were examined.

#### 3.3.4. Non-Initiation of GAHT

Finally, we examined the reasons why 62 transgender adolescents in our cohort had not initiated GAHT by the end of the data-collection period. Twelve (19.4%) of them had not reached the team’s minimum required age of 15 years to receive GAHT. For the others, 19 of them (30.6%) had insufficient follow-up, meaning that their care with the team had only recently begun by the end of the inclusion period and/or they had not yet had an endocrinology consultation by that date. Three (4.8%) adolescents did not appear to meet the required maturity criteria, either because of irregular follow-up, repeated changes of opinion, or the presence of a major depressive episode in which gender identity was not considered to be the primary source of psychological distress. In such cases, the team chose to allow additional time for reflection. For 7 (11.3%) adolescents, the decision to postpone GAHT reflected a family level choice to allow more time for reflection. This differs from the strict refusal of one or more holders of parental authority, which concerned 7 adolescents too (11.3%). The other reasons are summarized in Table 4.

Treatment discontinuation and retransition.

Some adolescents who had not yet started treatment:

- Retransitioned to a gender aligned with their sex assigned at birth before meeting an endocrinologist *;
- Retransitioned to a gender aligned with their sex assigned at birth after meeting an endocrinologist but before initiating GAHT *.

Some adolescents who had started treatment:

- Interrupted GAHT or GnRH agonist due to an injection-related phobia but continued to identify as transgender *;
- Temporarily paused GAHT to continue their personal reflection, before later deciding to resume treatment *;
- Permanently discontinued GAHT without returning to a gender identity aligned with their sex assigned at birth, instead identifying with a less binary gender *.

No adolescent who had initiated GAHT subsequently retransitioned to a gender aligned with their sex assigned at birth by the end of the data collection period.

* These five categories represent a total of 7 adolescents (6.1% of the cohort). Exact numbers for each category are not provided due to the very small sample size, in order to preserve patient anonymity.

## 4. Discussion

### 4.1. Referral Dynamics

As in other cohorts [8,24,25,26], first presentations were initially rare and increased over time. The follow-up remains too limited to assess whether a plateau has been reached. The rise in care-seeking remains poorly understood and is likely multifactorial. It appears to depend partly on the expansion of available services, since the increase occurs at different times across countries, and partly on the likely broader dissemination of information [27]. Another hypothesis could be a growing societal acceptance of gender transition.

### 4.2. Population

The majority of adolescents in our cohort were AFAB (68.7%). This predominance is consistent with findings from other studies on transgender youths [8,28,29]. This overrepresentation of AFAB adolescents is one of the points of controversy in the care of transgender minors. However, international literature suggests that although the age at which hormonal interventions are initiated may differ according to sex assigned at birth, the sex ratio tends to balance out over time [19,28,30,31].

Several hypotheses have been proposed to explain this phenomenon:

- The difference in the timing of puberty between sexes. Among AFAB adolescents, the development of secondary sex characteristics occurs physiologically earlier, which may lead to an earlier intensification of psychological distress.
- Another hypothesis is a greater societal acceptability AFAB transition compared with AMAB individuals who appear to be exposed to higher levels of social intolerance [28,30].
- Another study [31] found that, among sexual minorities, individuals AMAB (cis or trans) experience disproportionately greater difficulty disclosing their identity during adolescence compared with those assigned female at birth.
- Finally, AFAB adolescents appear to present higher rates of psychiatric co-occurring conditions and school disengagement, as well as a more frequent social transition, which may lead them to seek care earlier [8].

The ages of coming in and coming out were self-reported by adolescents during consultations. These variables are therefore subjective, depending both on the adolescent’s memory and on their personal interpretation of their gender incongruence. Similarly, coming out may represent a full social transition for some, while for others it may correspond only to a private conversation with a friend and/or a family member. This explains the substantial variability observed. The median age of coming in in our study was 12 years old (Q1: 10; Q3: 14), and the interval between the reported coming in and the first consultation with the team was 3 years (Q1: 1; Q3: 5). This suggests that personal reflection prior to any transition-related steps is a long process, often unfolding over several years.

### 4.3. Mental Health

We compared several psychiatric co-occurrences and psychosocial factors in our cohort with those of the general population [18,20,21,22,23,32,33] and with the findings reported by the only other French cohort [8]. In our cohort, the proportions of anxiety disorders, depressive syndromes, suicidal ideation, and suicide attempts were significantly different from those observed in the general population. However, no significant difference was found for eating disorders, whose prevalence was comparable to that of the general population.

Our results are comparable to those reported by the other French cohort [8] and are consistent with the international literature on this topic [34]. However, we note that our rates of depressive syndromes are lower and our rates of ADHD a bit higher than those observed by Lagrange et al. [8]. We do not have a clear explanation for this discrepancy. The retrospective nature of our study may have led to an under-identification of depressive disorders in some patients. Nevertheless, the proportions observed in both cohorts remain higher than those reported in the general population.

In our cohort, the prevalences of ASD and ADHD were significantly different from those estimated in France [22,23]. Numerous studies [8,35] have already highlighted the association between ASD and gender incongruence. A systematic review focusing specifically on the relationship between autism and gender diversity [35] proposed several hypotheses to explain this association. One of them suggests that difficulties in perceiving social norms, characteristic of autism, may lead to reduced identification with gender norms and reduced pressure to conform to them. This could result in a higher and earlier rate of transgender coming out among adolescents with ASD.

Similarly, ADHD also appears to have a higher prevalence among transgender populations, both in our study and in the literature [34]. The causal nature of this association remains undetermined.

School bullying was another variable we considered relevant in our study. It affected 29.6% of adolescents in our cohort, compared with 4 to 6% of students in France [20]. The retrospective design of our study did not allow us to determine whether bullying was consistently related to gender identity. These results nevertheless suggest an increased vulnerability among the adolescents in our cohort to this phenomenon, which is consistent with the existing literature [36].

### 4.4. Type of Schooling Impact

In France, the vocational track corresponds to a more practical, profession-oriented curriculum. Students spend only part of their time in school (often about half, sometimes less), while the remaining time is spent working in a company under the supervision of an employer who trains them in their future profession. They are paid for this work and therefore have a mixed status, both as students and employees.

Surprisingly, in our cohort, transition pathways appear to differ according to the adolescent’s type of schooling. Adolescents enrolled in non-general education tracks (vocational track, remote learning, civic service, employed, inactive, and/or had dropped out of school) seemed more likely to consult an endocrinologist than those in general education tracks. They also consult more often an adult endocrinologist than a pediatric one. To our knowledge, these parameters have not been examined in other studies. Given the substantial variability in educational systems across countries, international comparison is challenging. It would therefore be valuable to explore in dedicated studies how these parameters influence endocrine support. One hypothesis is that transgender adolescents enrolled in vocational programs or apprenticeships may be confronted earlier with adult responsibilities and professional environments, leading to earlier autonomy and a more assertive decision-making process regarding their transition.

### 4.5. Practical Aspects of the Support Provided

The median duration of follow-up before initiating GAHT was 15 months (Q1: 11 months; Q3: 21 months) in our cohort, with a mean age of 16.99 years (±0.98). The overall proportion of adolescents receiving GAHT in our cohort was 46%, and only 34.8% among those were under 18 at initiation. These figures are comparable to those in previous cohorts [8,11,12]. These findings imply that more than half of adolescents consulting for gender incongruence do not pursue hormonal transition, at least not before age 18. For those who do, hormone therapy represents the culmination of a long process: from coming in to coming out, followed by medical assessment, and finally hormonal transition.

Only 4.3% of adolescents in our cohort received a GnRH agonist. This proportion varies considerably (from 11% to 98%) between studies [8,11,12,15,16], likely due to local organizational and study design differences. For example, the other French cohort reported 11% on puberty blockers, but it was the only local network offering such care since 2013, whereas our network opened a dedicated pediatric endocrinology clinic only in 2020. Prior to that, puberty blockers were not prescribed for younger adolescents in our network.

### 4.6. Fertility

Before initiating GAHT, an informational consultation at the fertility preservation center was systematically proposed but only a small proportion of adolescents actually attended it (27.8%). Nevertheless, about half of those who initiated GAHT had attended an informational consultation, suggesting this issue becomes more salient as GAHT approaches. Only 11% of adolescents on GAHT underwent gamete preservation, and none were AFAB. These dates are consistent with the literature [8,37] and may be explained by:

- The greater complexity of oocyte preservation compared with sperm preservation (even though sperm preservation is also often experienced negatively by adolescents with gender dysphoria, for whom the requirement to masturbate may be psychologically intolerable).
- The time of this request that arises when adolescents are fully engaged in their transition process, generally wishing to avoid any additional delays and not always having the maturity or the necessary perspective to consider future fertility issues or potential parenthood [37].
- The specific challenges posed by fertility preservation for adolescents on puberty blockers, as these patients do not yet produce gametes.
- Regulatory barriers that further complicate the use of preserved gametes in adulthood. In France, gamete preservation is entirely free of charge and fully covered by the national healthcare system. Nevertheless, transgender individuals cannot currently use their stored gametes if they have legally changed their sex on civil records. Some adolescents therefore prefer not to preserve gametes that they may never be able to use.

This combination of factors likely contributes to the high rate of refusal of consultations and, consequently, of fertility preservation procedures. Finally, it should be noted that, to our knowledge, no study has assessed how many individuals ultimately use of the gametes they preserved.

### 4.7. Absence of Treatment, Treatment Discontinuation, and Retransition

We also examined the reasons why some adolescents did not receive hormone therapy. Three (4.8%) adolescents did not appear to meet the maturity criteria required by international recommendation [1]. However, this maturity criterion raises questions regarding the subjective interpretation it entails and the tools used to assess it. Maturity can be defined in various ways, both within and between individuals, underscoring the importance of coordinated multidisciplinary work.

For 13% of adolescents, the reason was a family-driven period of reflection, which does not constitute opposition to hormonal transition but rather a desire for discussion or postponement. This differs from the strict refusal of one or more holders of parental authority, which concerned seven adolescents (11%) in our study.

Finally, we sought to examine retransition/detransition within our cohort. Definitions of retransition vary widely across studies [38,39,40]. Depending on which criteria are applied among the different categories, the rate of retransition/treatment discontinuation in our study ranges from 0% (with no adolescent returning to their sex assigned at birth after initiation of GAHT) to 6.1% (when combining several criteria). This aligns with the broad range reported in the literature, where figures vary substantially from one study to another. It is nonetheless reassuring that no adolescent in our cohort retransitioned to their sex assigned at birth after starting GAHT by the end of data collection. This suggests that each case underwent rigorous prior evaluation by our team. However, it should be noted that these assessments are sometimes experienced as too long and perceived negatively by some patients. A future reassessment of our cohort may be useful to evaluate how the proportion of retransition evolves over time.

Moreover, retransition often concerns individuals who are subsequently lost to follow-up, as they no longer consult teams specializing in transgender care, which makes it difficult to obtain reliable data. In our Strasbourg cohort, a number of adolescents (not treated with GAHT) spaced out their appointments or discontinued follow-up before endocrine support could be initiated. Their exact number is difficult to determine due to the retrospective nature of our study and the possibility that adolescents may choose another care team once they reach adulthood. For these individuals, the reasons for discontinuing follow-up are unknown. Some may no longer experience gender incongruence, in which case our retransition rate would be underestimated for adolescents who did not receive hormonal treatment.

A literature review [39], including 15 observational studies, reported point prevalences of retransition ranging from 0.8% to 7.4% before treatment, and from 1% to 9.8% after treatment initiation. The author highlighted considerable heterogeneity in definitions of detransition and in conceptual frameworks across studies. Our findings clearly support this observation. Harmonizing the definition of retransition would therefore be valuable to obtain comparable and reliable estimates for this patient population.

## 5. Conclusions

Although hormonal treatment for transgender adults has been available for several decades, medical support for minors is more recent, with increasing numbers of requests, particularly among adolescents, generating controversial debates and reflections across political, social, and medical spheres. Social transition among young people is becoming increasingly accepted, reflecting a societal shift toward a less binary understanding of gender. However, medical transition in minors remains governed by recommendations that are often unclear, constantly evolving, or, in many countries, entirely absent.

In France, the consensus statement of the French Society of Pediatric Endocrinology and Diabetology is recent, following an expert report published in November 2024. Official recommendations from the French National Authority for Health were published in 2025 but currently exclude minors from their scope. Developing such guidelines is challenging, largely because transgender children and adolescents remain understudied, given the relatively recent emergence of structured medical pathways. Their trajectories and long-term outcomes are poorly documented, and the number of minors concerned is not systematically recorded.

This observation highlights the value of network-based care, which appears to be the direction taken by French professionals at both local and national levels. Since the end of our data collection, multidisciplinary discussions have evolved, and the Strasbourg network is now part of a regional one involving five university hospitals (Reims, Nancy, Strasbourg, Dijon, and Réunion Island) and their local network. This structure allows for more frequent meetings with the aim of improving territorial coordination, harmonizing care pathways, and developing a broader understanding of the population concerned.

As in several other scientific publications, we observed a substantial proportion of adolescents experiencing psychological distress and/or presenting neurodevelopmental differences. This finding prompted the reflection on how best to support these adolescents and thereby reduce suicidality and other psychiatric co-occurrences within this population. We also found, consistent with other studies, a predominance of AFAB adolescents, raising questions about the barriers that AMAB individuals may face when seeking to transition, given that the sex ratio among transgender adults appears to approach parity.

In our cohort of 115 adolescents, approximately 45% received hormone therapy, and only 35% initiated treatment before age 18. These figures help put into perspective the notion of a supposed “epidemic” of pediatric transgender identity sometimes invoked in political or media discourse. They also highlight that hormonal treatment is neither immediate nor inevitable in the care pathways of these adolescents, more than one-third of whom never meet an endocrinologist. Unexpectedly, our study revealed an impact of school type on transition pathway. This result would warrant further investigation in a dedicated study.

Finally, we showed that depending on the definition applied, the retransition rate could vary from 0% to 6.1%, once again raising questions about the validity and comparability of studies on retransition. This topic would benefit from being addressed by international scientific societies in order to establish one or more shared definitions according to the type of retransition considered.

In conclusion, this work represents the second French pediatric cohort of transgender minors. Future research should involve larger-scale studies to compare care pathways and, we hope, to shed light on how to better support the distress experienced by these adolescents and improve overall care. In the meantime, and given the current political climate, structuring and coordinating all actors involved, both in Strasbourg and across the country, appear essential to ensure that the care offer is as clear and accessible as possible for adolescents, their families, and public authorities. It is equally important to promote academic research on this topic and to accumulate prospective data that will help improve the overall health of this population and, we hope, reinforce the legitimacy of these care pathways.

## Figures and Tables

**Table 1:** Psychiatric co-occurrences and other relevant medical history.

Psychiatric Co-Occurrences	CIM10	*n* = 115
Anxiety disorder	F41	60 (52.1%)
Self-harm behavior	Z915, X78	42 (36.5%)
Suicidal ideation	R45.81	72 (62.6%)
Suicide attempts	X60 to X84	33 (28.7%)
Depressive disorder	F32.9	39 (33.9%)
Eating disorder	F50	17 (14.8%)
Social and school phobias	F40.1	10 (8.7%)
Sleep disorder	F51	12 (10.4%)
Behavioral disorder	F98.9	2 (1.7%)
Sensory processing disorder	R44.8	1 (0.8%)
Obsessive compulsive disorder	F42	2 (1.7%)
Personality disorder	F60	3 (2.6%)
Bipolar affective disorder	F31	1 (0.8%)
Schizophrenia	F20	1 (0.8%)
Substance misuse	Z86.4	4 (3.5%)
Intellectual disability	F79	1 (0.8%)
Dyslexia/dyscalculia/dysorthographia/dysgraphia	F81	3 (0.02%)
Autism spectrum disorder	F84	9 (7.8%)
Attention deficit/hyperactivity disorder	F90	15 (13.0%)
**Associated Psychosocial Factors**		
History of sexual violence	Z91.4; Z61.5; T74.3; Z61.4	12 (10.4%)
School bullying	Z60.4	34 (29.6%)

**Table 2:** Comparison of mental health figures in our cohort with the general population (in France) and with the data from the cohort of Lagrange et al. (another French cohort) reported in the literature [18,19,20,21,22,23].

	*n* = 115	General Population (References)	OR [CI 95%]	Lagrange et al. Cohort *n* = 239	OR [CI 95%]
School bullying	34 (29.6%)	6.0% ([20])	**6.6 [4.4–9.9]**	38%	0.7 [0.4–1.1]
Anxiety disorder	60 (52.1%)	14% ([18])	**6.9 [4.8–10]**	41%	1.6 [1–2.5]
Suicidal ideation	72 (62.6%)	24% ([18])	**5.3 [3.6–7.8]**	46%	**2 [1.2–3.1]**
Hospital admission for suicide attempts	33 (28.7%)	2.8% ([18])	**14 [9.2–21.3]**	24%	1.3 [0.8–2.1]
Depressive disorder	39 (33.9%)	14% ([18])	**3.2 [2.1–4.7]**	60%	**0.3 [0.2–0.5]**
Eating disorder *	17 (14.8%)	No official data	/	11%	1.4 [0.7–2.7]
Autism spectrum disorder	9 (7.8%)	1.0% ([21,22])	**8.4 [4.3–16.6]**	9%	0.9 [0.4–1.9]
Attention deficit/hyperactivity disorder	15 (13.0%)	3.7% ([19,23])	**3.9 [2.2–7]**	6%	**2.4 [1.1–5.0]**

* Figures for anorexia nervosa and bulimia are combined here. The bolded results correspond to the odds ratios whose confidence intervals do not include 1.

**Table 3:** Data summarizing the medical management of transgender identity in our cohort (percentages calculated based on the total cohort size).

Hormonal Management	Results
Initial Consultation with the Endocrinologist	
Mean age (standard deviation)	16.04 (1.87)
AFAB (*n*)	53 (46%)
AMAB (*n*)	22 (19%)
Pediatric endocrinologist (*n*)	40 (35%)
Adult endocrinologist (*n*)	35 (30.4%)
Follow-Up Prior to Treatment	
Time (median with quartile) since first contact with the team, in months	15 (Q1:11; Q3:22)
Time (median with quartile) since first contact with the endocrinologist, in months	4 (Q1:2; Q3:10)
Number (median with quartile) of child psychiatry consultations prior to GAHT	9 (Q1:7; Q3:12)
Number (median with quartile) of endocrinology consultations prior to GAHT	2 (Q1: 2; Q3:3)
Fertility	
Fertility preservation center consultation (*n*)	32 (27.8%)
Fertility preservation center consultation prior to GAHT (*n*)	26 (22.6%)
Gamete preservation (*n*)	6 (5.2%)
Multidisciplinary Team (MDT) Meeting for GAHT	
Case discussed at MDT (*n*)	33 (28.7%)
At least one unfavorable opinion at prior MDT (*n*)	2 (1.7%)
Hormone Therapy	
Mean age (standard deviation) at initiation of gender-affirming hormone therapy (GAHT)	16.99 (0.99)
GAHT (*n*)	53 (46%)
Puberty blocker (*n*)	5 (4.3%)

**Table 4:** Reasons for the absence of initiation of gender-affirming hormone therapy (GAHT).

Absence of GAHT by the End of the Data Collection Period Because (*n* = 62):	*n*	*%*
Age < 15 yo	12	19.4%
Age ≥ 15 yo, including:	50	80.6%
Refusal by at least one holder of parental authority	7	11.3%
Family deliberation ongoing	7	11.3%
Personal deliberation regarding GAHT ongoing	4	6.5%
Insufficient follow-up by the medical team	19	30.6%
Maturity criterion non met	3	4.8%
Somatic contraindication	0	0%
Unfavorable opinion at MDT meeting	0	0%
Patient on puberty blocker with GAHT not yet initiated	1	1.6%
Change of mind with social retransition, medical transition not initiated	2	3.2%
Gender Fluid	1	1.6%
Other	4	6.5%
Not documented	2	3.2%

## Data Availability

The data presented in this study are available on request from the corresponding authors. The data are not publicly available due to privacy and ethical reasons.

## Abbreviations

ADHD	Attention-Deficit/Hyperactivity Disorder
AFAB	Assigned Female At Birth
AMAB	Assigned Male At Birth
ASD	Autism Spectrum Disorder
CI 95%	95% confidence interval
GAHT	Gender-affirming hormone therapy
ICD	International Classification of Diseases
MDT	Multidisciplinary Team meeting
OR	Odd ratio
WPATH	World Professional Association for Transgender Health
